# Changes in Oral-Health-Related Quality of Life of Egyptian Children Treated under Dental General Anesthesia: A Prospective Study

**DOI:** 10.3390/jcm12185792

**Published:** 2023-09-06

**Authors:** Mahmoud Faheem, Dalia Moheb, Sherif Bahgat, Christian Splieth, Katrin Bekes

**Affiliations:** 1 Department of Preventive and Pediatric Dentistry, University of Greifswald, 17489 Greifswald, Germany; mahmoudfaheem@outlook.com (M.F.); splieth@uni-greifswald.de (C.S.); 2 Department of Pediatric Dentistry and Dental Public Health, Faculty of Dentistry, Cairo University, Cairo 12613, Egypt; dr.daliamoheb@hotmail.com (D.M.);; 3 School of Dentistry, Newgiza University, Giza 12577, Egypt; 4 Department of Paediatric Dentistry, University Clinic of Dentistry, Medical University of Vienna, 1090 Vienna, Austria

**Keywords:** oral-health-related quality of life, dental general anesthesia, early childhood oral health impact scale

## Abstract

Background: Treatment of young children under dental general anesthesia (DGA) is sometimes necessary due to lack of cooperation and the complexity of dental treatment. The aim of this study was to assess the changes in oral-health-related quality of life (OHRQoL) in children following treatment under DGA. Methods: A consecutive sample of 88 children aged 5 and younger who were referred to the department of pediatric dentistry, Cairo university, Egypt, for treatment under DGA was included. Parents were asked to complete the Arabic version of the Early Childhood Oral Health Impact Scale (A-ECOHIS) questionnaire before and 4 weeks after treatment. The Wilcoxon signed-rank test was used to compare baseline and follow up scores. Effect sizes (ES) were also calculated. Results: The overall ECOHIS scores decreased significantly from 16.72 (±7.07) to 0.9 (±3.08); (*p* < 0.001, Wilcoxon signed-rank test) after treatment under DGA, demonstrating a large effect size of 2.2. The scores of the two subscales of the ECOHIS, the child impact scale (CIS) and the family impact scale (FIS), also decreased significantly (*p* < 0.001). Conclusions: Treatment under DGA not only improved the OHRQoL of the Egyptian children in our sample significantly, but also had a positive effect on their families’ quality of life.

## 1. Introduction

Dental caries is still the tenth most prevalent condition worldwide, affecting 621 million children [1]. Although most children are able to receive dental treatment in a conventional setting, some patients fail to respond to the usual behavior management techniques and must therefore be treated under dental general anesthesia (DGA) [2]. This places a considerable burden on health systems. The prevalence of dental caries in Egypt is very high in international terms [3]. In 2014, a nationwide survey that covered individuals from Egypt’s governorates showed that nearly 70% of examined children had some untreated caries experience [4]. This study was the first nationwide survey to collect comprehensive information on the status of oral health among Egyptian adults and children since the last survey conducted over 23 years ago. It covered over 10,000 individuals from Egypt’s 27 governorates and involved different sectors of society, including orphanages and prisons. Another study, which assessed caries risk using the caries management by risk assessment (CAMBRA) protocol among Egyptian children aged 3–12 years, concluded that caries risk was high among these children [5]. The impact of dental caries and DGA treatment on the quality of life of Egyptian children has not yet been studied.

Recently, there has been a move to evaluate patient-reported outcomes along with the clinical outcomes of medical treatments. Oral-health-related quality of life (OHRQoL) is an important measure to assess the impact of oral health on patients’ daily life [6]. For children of preschool age and younger, the ECOHIS (Early Childhood Oral Health Impact Scale) is the most commonly used, widely translated, and validated instrument [7,8,9]. Furthermore, it has also been found to be sensitive and responsive to DGA treatment effects [10,11]. Many studies have shown the positive impact of dental treatment under DGA for these children and the wide acceptance for this treatment modality by their parents [12,13].

Currently, there are no available data from studies investigating the effect of dental treatment under DGA for preschool children in the Egyptian population. Therefore, the aim of our study was the analysis of changes in OHRQoL of children aged up to five years in Cairo following dental treatment under DGA. The research hypothesis for our study was the assumption that the dental treatment under DGA for children under the age of five years in Egypt would positively affect the oral-health-related quality of life of the children belonging to this age group.

## 2. Materials and Methods

For this prospective clinical study, ethical approval was obtained from the ethical committee at the Faculty of Dentistry, Cairo University, Egypt. All children at the age of 5 years old or younger who were in need of dental treatment under DGA and presented at the Department of Pediatric dentistry, Cairo University, Egypt, during the eight-month recruiting phase (between December 2017 and August 2018) were included in this study. The study was voluntary, and the children’s parents or their legal guardians provided their written informed consent. Children with any form of disability or medical condition or taking any kind of medications that may affect their quality of life were excluded, as were those with incompletely filled questionnaires. The obtained data comprised personal background, medical conditions, and clinical dental examinations. Therein, personal background data included age, gender, and migrational backgrounds. The Arabic version of the Early Childhood Oral Impact Scale (A-ECOHIS) questionnaire was used to assess the OHRQoL before and four weeks after DGA. Two additional Global Transition Judgment Questions (GTJQs) were added to the original ECOHIS questionnaire, with the first one assessing the general wellbeing of the child (“How would you rate the general health status of your child?) and the second (“How would you rate the health of your child’s teeth, jaws, and mouth?”) assessing the oral health of the child. The indications for referral for DGA along with the different treatments delivered to these children were also recorded.

All data were entered primarily into predesigned Excel sheets and then transferred to SPSS (version 21.0: SPSS Inc., Armonk, NY, USA) for Windows to perform the statistical analysis. Descriptive analysis using means, distributions, and standard deviations (SD) was carried out to describe the characteristics of treated patients and the indications for the referral along with the treatments performed for those patients under DGA. The A-ECOHIS data were also analyzed using the same program. Questionnaires with incomplete responses were excluded from the analysis. The Wilcoxon signed-rank test was used to compare the baseline and follow up scores and test the statistical significance of the changes. The level of the statistical significance was set at 95% (*p* = 0.05) in all the analyses. Effect sizes (ESs) were calculated for each domain by deducting the postoperative score from the pre-operative score and dividing the difference by the standard deviation of the pre-operative score [11]. These (ESs) indicated the magnitude of change. An ES <0.2 indicated a small change, an ES of 0.2–0.7 indicated moderate change, and an ES of >0.7 indicated a large change [11].

## 3. Results

### 3.1. Sample Description

One hundred and twenty patients treated under DGA were recruited for this study. Of these, twenty-two patients had to be excluded as they were found to be medically compromised, suffering from a general disease and/or disability that may affect their quality of life. Moreover, ten questionnaires were found to be incompletely filled in and were excluded as well. The remaining data of 88 children were then eligible for statistical analysis.

The children included in this study were 42 girls and 46 boys with a mean age of 3.80 (±1.11) years. They were Egyptians, with no patients with an immigration background recorded. The dental index of decayed, missing, and filled teeth (dmft) recorded for each patient before initiating the dental treatment was found to have a mean score of 9.74 (±3.41), with the highest recorded score being 16.

### 3.2. Indications of Treatment under DGA

The most common single reason for referring patients to be treated under DGA in our study was the “lack of child cooperation” (92.2%), followed by “dental fear or apprehension” (51.1%).

### 3.3. Changes in OHRQoL after Treatment under DGA

The overall ECOHIS scores decreased significantly (*p* < 0.001, Wilcoxon signed-rank test) after treatment under DGA from 16.72 (±7.07) to 0.9 (±3.08), representing a 94.6% change and demonstrating a large effect size of 2.2. The scores of the two subscales of the ECOHIS, the child impact scale (CIS) and the family impact scale (FIS), also decreased significantly (*p* < 0.001) from 9.92 (±4.94) to 0.57 (±1.94) and from 6.80 (±3.77) to 0.33 (±1.37), representing a 94.6% and 95.1% change from before to after DGA, respectively. In the CIS, the greatest decreases were found in the domains of “child self-image” and “child psychology” (100% and 97.4%, respectively), whereas the domain of “parental distress” showed the greatest decrease in the FIS, at 96.2%. All scores of all items/questions of the CIS and FIS showed a significant decrease (*p* < 0.001, Wilcoxon signed-rank test), demonstrating large effect sizes except for the items assessing “child self-image” (*p* = 0.008), showing a medium effect size (Table 1).

The prevalence of the most frequently reported child and family impacts at baseline and post-treatment are shown in Table 2. At baseline, “pain”, “eating problems”, “trouble sleeping”, and “being irritable” were the most frequently reported impacts in the CIS, while parents “feeling guilty” and the “financial impact” on the family were the most common impacts reported in the FIS. On the other hand, “pain”, the “financial impact” on the family, “eating problems”, and parents “feeling guilty” were the most frequently reported impacts by parents at the post-treatment follow up appointment. The largest decrease in prevalence after dental treatment under DGA was found in the items of the “financial impact” on the family, “eating problems”, “pain”, and parents “being upset”.

### 3.4. Global Transition Judgment Questions (GTJQs)

Baseline and post-treatment scores representing the parents’ response to the additional GTJQs are presented in Table 3. The scores of both questions increased significantly (*p* < 0.001, Wilcoxon signed-rank test) postoperatively, denoting improvement in the parents’ perception of the general and oral health of their children after DGA.

After treatment under DGA, 40% of the respondents reported an improvement in the general health and wellbeing of their children, whereas 58% perceived no change. Furthermore, 84% of the participants reported that their children’s OHRQoL, indicated by the GTJQ regarding their oral health, improved after treatment under DGA, whereas 13.6% reported no change compared to before initiating treatment. Only two participants reported deterioration in OHRQoL on the global scale post-operatively.

### 3.5. Items of the A-ECOHIS

Parents’ detailed responses (at baseline and post-treatment) to different items of the A-ECOHIS are presented in Table 4, which illustrates the change seen in parents’ ratings of their children’s general oral health (teeth, jaws, and mouth) following treatment under DGA, with most of them (96.6%) scoring good, very good, and excellent in comparison to only 51.1% preoperatively.

## 4. Discussion

Although patients suffered poor OHRQoL before treatment, it was proven in this study that this was significantly improved after treatment under DGA, which had a good impact on patients and their families as well. This is the first study assessing both treatments offered for children under DGA and the changes in OHRQoL after this treatment in the Egyptian population. Data assessing these changes in non-Arabic-speaking countries are abundantly available in the published literature [14,15], whereas available data from Arabic-speaking countries seem to be relatively fewer [16,17,18]. To the best of our knowledge, there are scarce well-documented data from Egypt describing different treatments carried out under DGA, the reasons for referral for this type of treatment, and the effect of this treatment on the children’s OHRQOL, whereas records from other Arabic-speaking countries such as Kuwait [19], Saudi Arabia [20], and the United Arab Emirates do exist [21]. The observed changes in OHRQoL in this study are consistent with other studies that also reported an improvement in OHRQoL after treatment under DGA [10,22,23].

The relatively small sample size in this study can be considered as a limitation that makes a generalization of the results to the whole Egyptian population difficult. A study with a larger sample that is distributed evenly among different areas in Egypt would be favorable. The absence of a control group due to ethical reasons and the exclusion of the medically compromised patients or patients taking any medications that may affect their OHRQoL are considered further limitations of this study. Assessing the post-treatment change in ECOHIS scores at the follow-up appointment after 4 weeks, although reported in many other studies [24,25], seems not to be completely reflective of the long-term changes in the OHRQoL of children. A study with more follow-up intervals (4 weeks, 36 months, 1 year) could give a closer and more precise idea about the sustainability of these improvements in the OHRQoL of children after treatment under DGA.

The response rate to the post-operative follow-up appointments in this study (89.8%) can be considered as a strength in comparison to other published studies [2,26,27]. The reasons for this were either failure to completely fill out the questionnaire or failure to attend the follow up appointment itself. In our study, the most frequent single reason for referring patients to be treated under DGA was the lack of child cooperation (92.2%), a finding that is also reported by Savanheimo et al., 2012, who reported that (65%) of the included patients were referred for the same reason [28]. These findings differ to those reported by other researchers, who found that the most frequently cited reason for referral to DGA was extensive dental decay and the need to perform multiple extractions [29]. This can be explained by the fact that the children in the sample in our study were in the age range of 0–5 years, during which the cooperation of the child with the dentist seems to be challenging.

For young patients, as in our study, a proxy (parent or caregiver) is needed to measure the changes in OHRQoL as they cannot fully nor properly express themselves or precisely assess their health status [26]. This was proven to be an acceptable method, as an agreement was found between caregiver and child reports of children’s OHRQoL [30]. Some studies used the parent–caregiver perceptions questionnaire (P-CPQ) to measure the change in the OHRQoL of children after treatment under DGA [18,24,25]. In our study, the A-ECOHIS was used to assess these changes after DGA treatment. The overall A-ECOHIS scores decreased significantly (*p* < 0.001) after treatment under DGA, representing a 94.6% change, demonstrating a large effect size. This was found to be higher than but consistent with the results of many other studies that reported significant improvements in the OHRQoL after DGA [8,31,32,33,34]. This improvement is not the effect of treatment under DGA per se, but mainly the effect of pain relief and discomfort, which theoretically can also be achieved using sedation as an alternative to DGA [23].

The scores of the two subscales of the ECOHIS, the child impact scale (CIS) and the family impact scale (FIS), also both decreased significantly in our study, representing a 94.6% and 95.1% change, respectively, denoting a large effect size. The effect sizes in this study were found to be higher than in other studies [31,35]. All scores of all items/questions of the CIS and FIS showed a significant decrease, demonstrating large effect sizes except for the items assessing child self-image, which demonstrated a medium effect size. The most frequently reported child and family impacts at baseline were found to be pain, eating problems, trouble sleeping, being irritable, parents feeling guilty, and the financial impact on the family. On the other hand, pain, the financial impact on the family, eating problems, and parents feeling guilty were the impacts most frequently reported by parents at the post-treatment follow up appointment. These findings show little differences to other studies [24,26,31]. It is difficult to compare studies assessing this type of dental treatment as the treatment scores are dependent on many factors such as the age of the child, type and complexity of treatments carried out, and the child’s general and oral condition before initiating treatment. The biggest decrease in prevalence after dental treatment under DGA was found in the items of the financial impact on the family, eating problems, pain, and parents being upset. The financial burden on the family as a result of the poor OHRQoL seems to be an important factor in a developing country, necessitating further investigation of the cost-effectiveness of the treatment under DGA.

This study reveals that dental treatment under DGA is a reliable and accepted method of therapy, specifically for the preschool children of this sample of the Egyptian population. The high costs and the complexity of such treatments as well as the high prevalence of caries in Egypt can motivate those who are interested in the field of pediatric dentistry in Egypt for further investigations considering the cost-effectiveness of such treatments. Well-designed, nationwide dental preventive programs for fighting caries including regular professional fluoridation and fissure sealants, as well as raising awareness of the importance of dental hygiene measurements and regular dental check-ups, should be broadened and supported. This study showed that the dental treatment under DGA significantly improved the OHRQoL of the Egyptian preschool children, which, in turn, would encourage the colleagues of pediatric dentistry specialists to consider this treatment modality in their treatment planning for such children.

## 5. Conclusions

Four weeks after treatment under dental general anesthesia, children suffering from early childhood caries showed significant improvements in their oral-health-related quality of life. Further studies must focus on the long-term effect of DGA and on the impact of different treatment options under DGA.

## Figures and Tables

**Table 1:** Overall Early Childhood Oral Health Impact Scale (ECOHIS), Child Impact Section (CIS), and Family Impact Section (FIS) scores pre-treatment and post-treatment for 88 children (0–5 years) treated under dental general anesthesia (DGA).

ECOHIS Domain (No. of Questions)	Pre-Treatment Mean (SD)	Post-Treatment Mean (SD)	*p*-Value	Mean Score Change (SD)	Effect Size (ES)
Overall (13)	16.72 (7.07)	0.9 (3.08)	<0.001	15.82 (7.00)	+2.2
Child impact scale (9)	9.92 (4.94)	0.57 (1.94)	<0.001	9.35 (4.75)	+1.9
Oral symptoms (1)	2.39 (1.17)	0.22 (0.73)	<0.001	2.17 (1.23)	+1.9
Function (4)	4.58 (3.44)	0.28 (0.96)	<0.001	4.30 (3.33)	+1.3
Psychology (2)	2.66 (2.31)	0.07 (0.33)	<0.001	2.59 (2.31)	+1.1
Self-image (2)	0.30 (0.95)	0.00 (0.00)	0.008	0.30 (0.95)	+0.3
Family impact scale (4)	6.80 (3.77)	0.33 (1.37)	<0.001	6.47 (3.80)	+1.7
Parental distress (2)	3.95 (2.60)	0.16 (0.74)	<0.001	3.80 (2.62)	+1.5
Family function (2)	2.84 (1.93)	0.17 (0.68)	<0.001	2.67 (2.03)	+1.4

**Table 2:** Prevalence of the most frequently reported impacts pre-treatment and post-treatment for 88 children treated under dental general anesthesia (DGA).

Item of the A-ECOHIS	Prevalence of Impacts Reported “Often” or “Very Often” ¹	
	Pre-Treatment n (%)	Post-Treatment n (%)
Pain in the teeth, mouth, and jaws	40 (45.5)	3 (3.4)
Difficulty drinking hot or cold beverages	27 (31.0)	0 (0)
Difficulty eating some foods	40 (45.5)	2 (2.3)
Difficulty pronouncing any words	6 (6.8)	0 (0)
Missing day-care, preschool, or school	13 (14.7)	0 (0)
Trouble sleeping	30 (34)	0 (0)
Being irritable or frustrated	25 (28.4)	0 (0)
Avoiding smiling or laughing	0 (0)	0 (0)
Avoiding talking	4 (4.5)	0 (0)
Parents being upset	36 (41)	0.0 (0)
Parents feeling guilty	35 (51.1)	1 (1.1)
Parents taking time off from work	18 (20.5)	0 (0)
Financial impact on the family	44 (50.0)	3 (3.4)

^1^ Values are the percentage of parents or caregivers reporting the impact ‘Often’ or ‘Very Often’.

**Table 3:** Baseline and post-treatment scores representing the parents’ responses to the GTJQs for children treated under dental general anesthesia.

GTJQ	Pre-Treatment Mean (SD)	Post-Treatment Mean (SD)	*p*-Value
1. General health and wellbeing	3.07 (0.71)	3.49 (0.59)	<0.001
2. Oral and dental health (teeth, jaws, and mouth)	1.45 (93)	3.22 (0.78)	<0.001

**Table 4:** Parents’ responses (at baseline and post-treatment) to different items of the A-ECOHIS.

Item of the ECOHIS		Baseline (Pre-Treatment) and Post-Treatment A-ECOHIS Scores Reported by Parents and Caregivers				
		Never No. of Patients (%)	Hardly Ever No. of Patients (%)	Occasionally No. of Patients (%)	Often No. of Patients (%)	Very Often No. of Patients (%)
Pain in the teeth, mouth, and jaws	Pre	5 (5.7)	15 (17)	28 (31.8)	21 (23.9)	19 (21.6)
	Post	80 (90.9)	1 (1.1)	4 (4.5)	2 (2.3)	1 (1.1)
Difficulty drinking hot or cold beverages	Pre	45 (51.1)	2 (2.3)	14 (15.9)	18 (20.5)	9 (10.2)
	Post	82 (93.2)	2 (2.3)	4 (4.5)	0 (0.0)	0 (0)
Difficulty eating some foods	Pre	28 (31.8)	4 (4.5)	16 (18.2)	22 (25)	18 (20.5)
	Post	81 (92)	1 (1.1)	4 (4.5)	2 (2.3)	0 (0)
Difficulty pronouncing any words	Pre	73 (83)	3 (3.4)	6 (6.8)	1 (1.1)	5 (5.7)
	Post	88 (100)	0 (0)	0 (0)	0 (0)	0 (0)
Missing day-care, preschool, or school	Pre	59 (67)	1 (1.1)	15 (17)	12 (13.6)	1 (1.1)
	Post	88 (100)	0 (0)	0 (0)	0 (0)	0 (0)
Trouble sleeping	Pre	40 (45.5)	6 (6.8)	12 (13.6)	26 (29.6)	4 (4.5)
	Post	86 (97.7)	0 (0)	2 (2.3)	0 (0.0)	0 (0)
Being irritable or frustrated	Pre	46 (53.2)	3 (3.4)	14 (15.9)	21 (23.9)	4 (4.5)
	Post	86 (97.7)	2 (2.3)	0 (0)	0 (0)	0 (0)
Avoiding smiling or laughing	Pre	84 (95.5)	0 (0)	4 (4.5)	0 (0)	0 (0)
	Post	88 (100)	0 (0)	0 (0)	0 (0)	0 (0)
Avoiding talking	Pre	80 (90.9)	3 (3.4)	1 (1.1)	3 (3.4)	1 (1.1)
	Post	88 (100)	0 (0.0)	0 (0.0)	0 (0.0)	0 (0.0)
Parents being upset	Pre	16 (18)	18 (20.5)	18 (20.5)	18 (20.5)	18 (20.5)
	Post	84 (95.5)	0 (0.0)	4 (4.5)	0 (0.0)	0 (0.0)
Parents feeling guilty	Pre	33 (37.5)	2 (2.3)	8 (9.1)	30 (34.1)	15 (17)
	Post	85 (96.6)	1 (1.1)	1 (1.1)	1 (1.1)	0 (0.0)
Parents taking time off from work	Pre	54 (61.4)	5 (5.7)	11 (12.5)	13 (14.8)	5 (5.7)
	Post	88 (100)	0 (0.0)	0 (0.0)	0 (0.0)	0 (0.0)
Financial impact on the family	Pre	31 (35.2)	2 (2.3)	11 (12.5)	36 (40.9)	8 (9.1)
	Post	82 (93.2)	1 (1.1)	2 (2.3)	2 (2.3)	1 (1.1)

## Data Availability

Restrictions apply to the availability of these data. Data were obtained from the department of pediatric dentistry, Cairo University, and are available from the authors with the permission of the department of pediatric dentistry, Cairo University.
